# Enhanced atherosclerosis in apolipoprotein E knockout rabbits: role of apoB48-rich remnant lipoproteins

**DOI:** 10.3389/fcvm.2024.1424064

**Published:** 2024-07-17

**Authors:** Manabu Niimi, Yajie Chen, Huanyu Zhao, Xiangming Tang, Fumikazu Matsuhisa, Huanjin Zhou, Haizhao Yan, Lu Chen, Shuji Kitajima, Akira Sato, Jianglin Fan

**Affiliations:** ^1^ Guangdong Province Key Laboratory, Southern China Institute of Large Animal Models for Biomedicine, School of Pharmacy and Food Engineering, Wuyi University, Jiangmen, China; ^2^ Department of Molecular Pathology, Interdisciplinary Graduate School of Medicine, University of Yamanashi, Chuo, Japan; ^3^ Department of Pathology, College of Basic Medical Sciences and The First Hospital, China Medical University, Shenyang, China; ^4^ Division of Biological Resources and Development, Analytical Research Center for Experimental Sciences, Saga University, Saga, Japan; ^5^ Key Laboratory of Regenerative Biology, South China Institute for Stem Cell, Biology and Regenerative Medicine, Guangzhou Institutes of Biomedicine and Health, Chinese Academy of Sciences, Guangzhou, China; ^6^ Department of Cardiology, Interdisciplinary Graduate School of Medicine, University of Yamanashi, Chuo, Japan

**Keywords:** apoB48, apoE, atherosclerosis, chylomicrons, lipoproteins, rabbits, remnant

## Abstract

**Introduction:**

Apolipoprotein E (apoE) acts as a binding molecule for both the low-density lipoprotein receptor and the lipoprotein receptor-related protein and this function is essential for facilitating the hepatocyte uptake of lipoproteins containing apoB. The absence of apoE leads to increased atherogenicity in both humans and mice, although the precise molecular mechanisms remain incompletely understood.

**Objectives:**

This study aimed to investigate the susceptibility of apoE knockout (KO) rabbits, in comparison with wild-type (WT) rabbits, to diet-induced hyperlipidemia and atherosclerosis.

**Methods:**

ApoE KO rabbits and WT rabbits were fed a diet containing 0.3% cholesterol for 16 weeks. Plasma lipid levels, lipoproteins, and apolipoproteins were analyzed. Atherosclerosis was evaluated at the endpoint of experiments. In addition, we evaluated the oxidizability of those lipoproteins containing apoB to investigate the possible mechanisms of atherosclerosis.

**Results:**

Male apoE KO rabbits showed significantly elevated levels of total cholesterol and triglycerides compared to WT rabbits, while female apoE KO rabbits displayed similar high total cholesterol levels, albeit with significantly higher triglycerides levels than WT controls. Notably, both male (2.1-fold increase) and female (1.6-fold increase) apoE KO rabbits exhibited a significantly augmented aortic lesion area compared to WT controls. Pathological examination showed that the increased intimal lesions in apoE KO rabbits were featured by heightened infiltration of macrophages (2.7-fold increase) and smooth muscle cells (2.5-fold increase). Furthermore, coronary atherosclerotic lesions were also increased by 1.3-fold in apoE KO rabbits. Lipoprotein analysis revealed that apoB48-rich beta-very-low-density lipoproteins were notably abundant in apoE KO rabbits, suggesting that these remnant lipoproteins of intestinal origin serve as the primary atherogenic lipoproteins. Moreover, apoB48-rich remnant lipoproteins isolated from apoE KO rabbits exhibited heightened susceptibility to copper-induced oxidation.

**Conclusions:**

The findings indicate that apoB48-rich remnant lipoproteins, resulting from apoE deficiency, possess greater atherogenic potential than apoB100-rich remnant lipoproteins, regardless of plasma TC levels.

## Introduction

Apolipoprotein E (apoE) is a glycoprotein with a molecular weight of 34 kDa. It is primarily synthesized in the liver but is also expressed in various other tissues including the adrenal gland, adipose tissue, brain, kidney, lung, macrophages, muscle, ovary, and spleen (1, 2). ApoE is contained in nearly all lipoproteins except for low-density lipoprotein (LDL). It serves as a ligand for both the LDL receptor and LDL receptor-related protein, enabling the liver to effectively uptake lipoproteins (3), particularly triglyceride-rich lipoproteins (TRL) like chylomicrons (CM), chyloremnants, very-low-density lipoprotein (VLDL), and intermediate-density lipoproteins (IDL) (4–6).

The patients with either apoE gene mutations or apoE deficiency develop the type III hyperlipoproteinemia, which exhibits mild hyperlipidemia but is predisposed to the premature development of atherosclerosis (7). Homozygote apoE deficient patients are characterized by having markedly high VLDL and IDL with accumulations of apoB48 and apoA-IV in VLDL, IDL, and LDL (8, 9). In general, one-third to one-half of type III hyperlipoproteinemia patients develop premature or accelerated atherosclerosis (10).

In addition to the liver, apoE is also produced by extrahepatic cells and has been proposed to exert multiple physiological functions (2). For example, apoE is expressed in monocyte-derived macrophages and protects against atherosclerosis independent upon plasma lipids (11, 12). ApoE expressed by adipocytes participates in regulating adipocyte size and cellular triglyceride metabolism (13, 14). In the central nerve system, apoE secreted by the astrocytes mediates lipid transport by forming high-density lipoprotein (HDL)-like lipoproteins in the cerebrospinal fluid (15). Moreover, apoE is possibly secreted from injured or stressed neurons, suggesting apoE may be involved in the tissue repair and protects neurons from excessive damage (1).

For the elucidation of apoE pathophysiological functions, several types of apoE knockout (KO) animals have been generated. Among them, apoE KO mice are best studied and widely used for cardiovascular research (16–18). ApoE KO mice showed hyperlipidemia even on a chow diet and exhibited severe hypercholesterolemia (>2,000 mg/dl) when on a Western-type diet while plasma triglycerides (TG) were minimally increased (17). They developed spontaneous atherosclerosis in the aorta on a normal chow diet and showed advanced lesions on a Western-type diet (18). While apoE knockout (KO) mice have been extensively utilized as an experimental tool for studying lipid metabolism and vascular disease, it is important to consider the limitations of translating their results directly to humans. There are the key differences between mouse and human lipoprotein metabolism (20). For example, mouse plasma lipoproteins are predominated by HDL due to the lack of plasma cholesteryl ester transfer protein (CETP), a critical mediator of lipoprotein neutral lipid transfer between HDLs and LDLs, which is abundant in humans (19). In addition, mouse apoB mRNA editing takes place not only in the intestine but also in the liver, resulting in the presence of apoB48 in all apoB-containing particles including CMs, VLDs, IDLs and LDLs (12), which is different from humans in which apoB mRNA editing was only present in the liver and apoB48 was exclusively contained in CMs and chyloremnants. The prevailing consensus is that the liver tends to catabolize apoB48-containing lipoproteins at a faster rate compared to apoB100-containing particles (21). Therefore, it is critical to investigate the apoE functions in lipoprotein metabolism using the models with human-like lipoprotein metabolism features (22, 23). Rabbit is one of the best models for the examination of human hyperlipidemia and atherosclerosis because rabbits possess CETP and intestinal apoB editing which like human but unlike mouse (24).

Previously, we created apoE KO rabbits using genome editing techniques and characterized the lipoprotein profiles (25). On a regular normal diet, apoE KO rabbits showed slightly higher plasma lipid levels than wild-type (WT) rabbits: TG (78 mg/dl in apoE KO vs. 33 mg/dl in WT) and total cholesterol (TC, 156 mg/dl in apoE KO vs. 24 mg/dl in WT) (25). However, it is important to note that mild hyperlipidemia on a normal diet does not induce atherosclerosis in apoE KO rabbits. To elucidate the functional roles of apoE in the pathogenesis of atherosclerosis, in the current study, we fed apoE KO and WT rabbits with a cholesterol-rich diet and performed the following experiment with two purposes in mind: (1) does apoE deficiency increase the susceptibility of atherosclerosis in KO rabbits? To address this question, we evaluated the extent of aortic and coronary atherosclerosis, as well as femoral atherosclerosis induced by balloon injury; (2) what are the mechanisms underlying apoE deficiency-induced atherosclerosis? We made extensive analyses of lipoproteins and apolipoproteins focusing on their biochemical features and biological properties.

Our findings revealed that absence of apoE in rabbits resulted in a significant increase in aortic atherosclerosis. Notably, this atherogenic effect was also observed independent of plasma TC levels in female apoE KO rabbits. The primary atherogenic lipoproteins in apoE KO rabbits are derived from the intestine and consist of apoB48-rich CM remnants. These lipoproteins exhibit increased susceptibility to oxidation, indicating that it is specifically these lipoproteins that contribute to enhanced atherogenicity *in vivo*.

## Materials and methods

### Rabbits

ApoE KO rabbits were produced and used as described previously (22, 25). Male and female homozygous apoE KO rabbits, as well as WT rabbits, were included in this experiment. ApoE KO rabbits with New Zealand white rabbit background were bred at Saga University and University of Yamanashi. WT New Zealand white rabbits were purchased from Kitayama Labes (Ina, Nagano, Japan). Rabbits were initially fed a normal regular diet (CR-3M; Clea Japan) until they reached 15 weeks of age. Subsequently, they were transitioned to a cholesterol-rich diet (CRD) with 0.3% cholesterol and 3% soybean oil (150 g/day) for a duration of 16 weeks.

### Ethics statement

All animal experiments conducted at the University of Yamanashi were approved by the Animal Care and Use Committee (Approval No. A3-50) and Saga University (Approval No. 28-049-0, 29-019-0).

### Examinations of plasma lipids, lipoproteins, and apolipoproteins

Blood were collected from the fasted animals through the intermediate auricular artery, and plasma was separated through centrifugation at 1,500 × g for 20 min at 4°C. Colorimetric assay kits were utilized to measure TC, TG, and HDL cholesterol (HDL-C) levels (FUJIFILM Wako Pure Chemical, Osaka, Japan) (26).

For lipoprotein analysis, plasma lipoproteins were separated through sequential density ultracentrifugation, following previously established protocols (27). The following seven density lipoprotein fractions were obtained: CMs and VLDLs with a density of less than 1.006 g/ml; IDLs with a density of 1.02 g/ml; LDLs with densities of 1.04 and 1.06 g/ml; and HDLs with densities of 1.08, 1.10, and 1.21 g/ml. TC and TG contents were measured in each lipoprotein fraction, and the apolipoproteins were analyzed using 4%–20% sodium dodecyl sulfate polyacrylamide gel electrophoresis (SDS-PAGE), followed by Coomassie brilliant blue (CBB) staining. Additionally, the fractions with *d* < 1.006 g/ml were further fractionated using 4% SDS-PAGE stained with CBB to analyze the contents of apoB100 and apoB48. The SDS-PAGE gels were scanned using a densitometer, and the optical density (OD) of apoB100 and apoB48 was measured using an image analysis system (Image J; National Institute of Health, Bethesda, MD).

### Electron microscopic analysis of apoB containing lipoproteins

ApoB-containing lipoproteins (*d* < 1.006, *d* = 1.02, 1.04, and 1.06 g/ml) were isolated from male and female rabbits fed a CRD, and analyzed by negative stain electron microscopy (28). The lipoproteins (50 μg/ml) on a formvar-coated grid were stained with 3% uranyl acetate. The micrographs were taken by a transmission electron microscope JEM-2100F (JEOL, Tokyo, Japan). Lipoprotein sizes on the micrographs were measured by an image analysis system (WinROOF; Mitani Corporation, Tokyo, Japan).

### Analysis of aortic and coronary atherosclerosis

A detailed protocol for analyzing rabbit atherosclerosis was previously described in a report (29). After 16 weeks of being fed CRD, the rabbits were euthanized by administering a lethal dose of sodium pentobarbital. The aortic trees were meticulously extracted and longitudinally opened, then secured onto a corkboard and fixed using 10% neutral buffered formalin. Sudan IV staining was performed on these aortae, and the Sudanophilic area was measured and quantified using WinROOF image analysis system (Mitani Corporation, Tokyo, Japan) (30). To assess microscopic lesions, the aorta was transversely cut at 4 mm intervals and embedded in paraffin. Thin sections of 3 μm thickness were obtained and subjected to staining with hematoxylin and eosin (HE), Elastica van Gieson (EVG), or immunohistochemical staining using monoclonal antibodies against macrophages (RAM11; Dako, Glostrup, Denmark) and smooth muscle α-actin (HHF35; Dako, Glostrup, Denmark). The microscopic lesions, as well as the areas positive for macrophages and smooth muscle cells, were quantified following previously established protocols (31). For the quantification of coronary atherosclerosis, the heart was divided into five blocks, and the left main coronary trunk was specifically used for analyzing lesion pathology and stenosis size. This analysis was performed using an image analysis system (24, 32).

### Balloon injury-induced femoral atherosclerotic lesions

In addition to aortic and coronary atherosclerosis, we evaluated the effect of apoE absence on the balloon injury-induced femoral atherosclerotic lesions. ApoE along with WT rabbits were given a CRD for 5 weeks and then, their right femoral arteries were subjected to endothelial injury by a balloon catheter as reported (33). In brief, the rabbits were first anesthetized with intravenous injections of ketamine (3 mg/kg) and medetomidine (0.06 mg/kg) and then maintained with isoflurane inhalation. A longitudinal incision in the right groin region was made to provide access to the saphenous artery. The right saphenous artery was incised, and a Fogarty 2F balloon catheter (Edwards Lifesciences, Irvine, CA) was introduced until it reached the abdominal aorta. The balloon was inflated by filling with 1 ml of saline and withdrawn once from the aortic bifurcation to the femoral artery (nearly 15 cm). The balloon was deflated and subsequently removed from the artery, followed by ligation of the saphenous artery. At 16 weeks, all the rabbits were euthanized as described above and femoral arteries were collected and fixed in 10% buffered formalin solution. The formalin-fixed femoral artery was cut at 4 mm intervals into six segments and embedded in paraffin and sectioned at 3 μm thickness. The serial sections were stained HE, and the intimal lesions were measured.

### Evaluation of lipoprotein oxidizability

The evaluation of lipoprotein oxidizability was conducted following previously established methods (34). ApoB-containing lipoproteins (*d* < 1.006, *d* = 1.02, 1.04, and 1.06 g/ml) were incubated with 30 μM CuCl_2_ in a microplate at 37°C for 300 min. The kinetics of the conjugate-diene production was determined by monitoring absorbance at 234 nm with an absorbance spectrophotometer (xMark; Bio-Rad Laboratories, Hercules, CA). Maximal oxidation speed (V max), maximal diene concentrations (OD max), and lag-time were calculated to estimate of oxidation sensitivity.

### Statistical analysis

The statistical analysis was performed using SPSS Statistics software (IBM Japan Ltd., Tokyo, Japan). The data are reported as either mean ± standard error of the mean (SEM) or standard deviation (SD). The normality of the data was assessed using the Shapiro-Wilk test. Parametric data were analyzed using either Student's *t*-test or Welch's *t*-test. Non-parametric data were analyzed using the Mann-Whitney *U*-test. A significance level of *P* < 0.05 was considered statistically significant.

## Results

### Analysis of plasma lipids and lipoproteins

**Figure 1:**
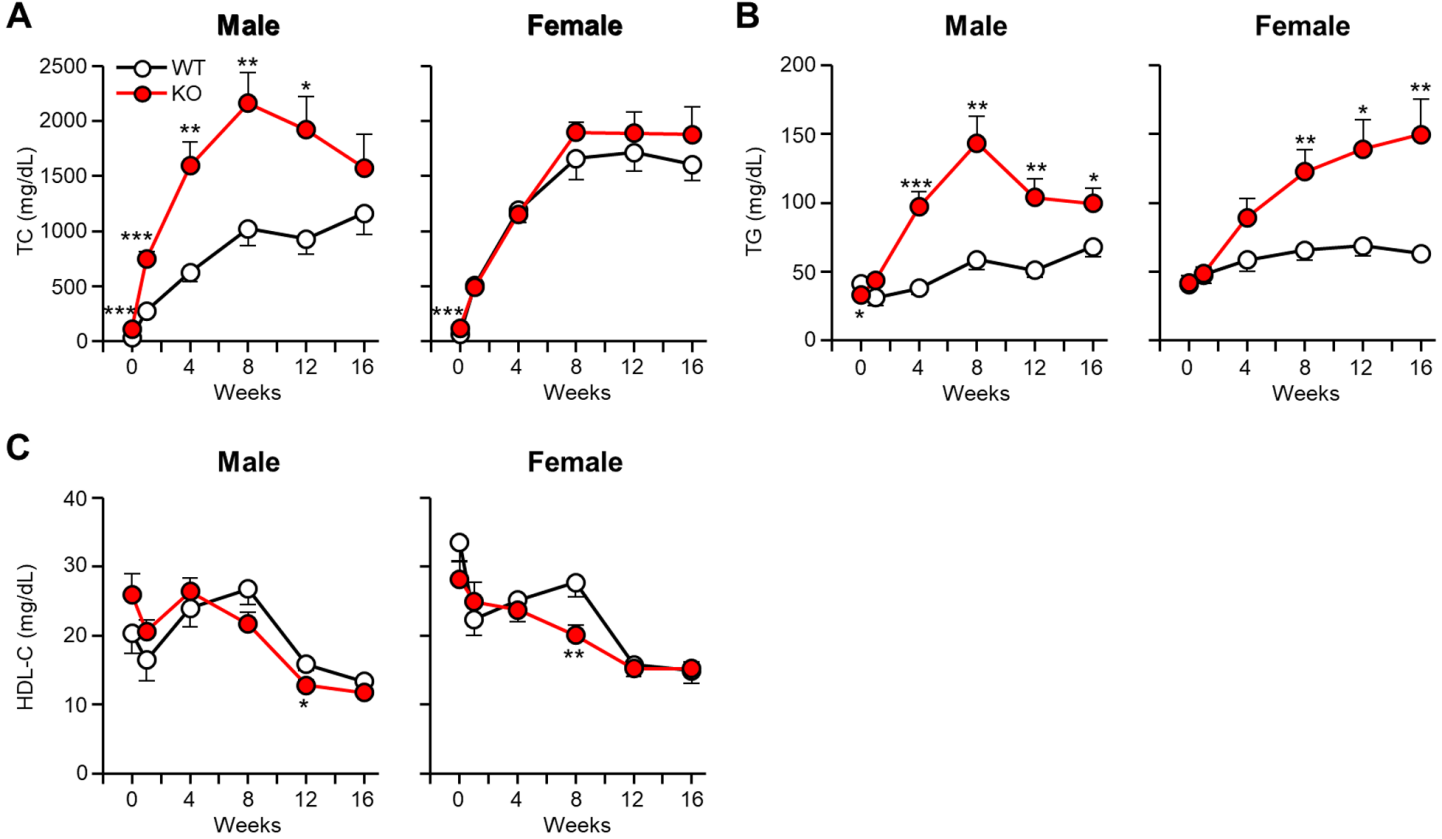
Analysis of plasma lipid levels after cholesterol-rich diet feeding. ApoE KO and WT rabbits were subjected to a 16-week cholesterol-rich diet. Monthly measurements were taken to determine the plasma levels of TC (**A**), TG (**B**), and HDL-C (**C**). The data are presented as mean ± SEM with a sample size of 8–10 per group. Statistical significance was denoted as follows: *p < 0.05, **p < 0.01, ***p < 0.001, when compared to the WT group.

As shown in Supplementary Figure S1, apoE KO rabbits showed significantly higher body weight than WT rabbits on a CRD for 16 weeks. CRD feeding resulted in the prominent increase of plasma levels of TC in all rabbits (Figure 1A). Interestingly, male apoE KO rabbits exhibited significantly “higher” hypercholesterolemia than WT rabbits, while female apoE KO rabbits showed almost “similar” hypercholesterolemia starting from 8 weeks (n.s.) to WT counterparts. These was further confirmed by measuring the values of area under the curve (AUC) of TC graphs. In the female rabbits, 21,920 ± 2,049 mg week/dl in WT vs. 23,971 ± 1,613 mg week/dl in apoE KO (*p* = 0.436) and in the male rabbits, 12,879 ± 1,795mg week/dl in WT vs. 26,584 ± 3,638 mg week/dl in apoE KO (*p* < 0.01).

Plasma levels of TG were significantly higher in apoE KO rabbits than WT rabbits (Figure 1B). AUC of TG in male KO rabbits were 2-fold greater than WT (792 ± 80 mg week/dl in WT vs. 1,633 ± 181 mg week/dl in apoE KO, *p* < 0.01) and 1.8-fold greater in female KO rabbits (984 ± 88 mg week/dl in WT vs. 1,776 ± 233 mg week/dl in apoE KO, *p* < 0.05). Plasma levels of HDL-C showed a tendency to decrease in all groups following CRD feeding. However, there was no significant difference observed between apoE KO and WT rabbits (Figure 1C).

**Figure 2:**
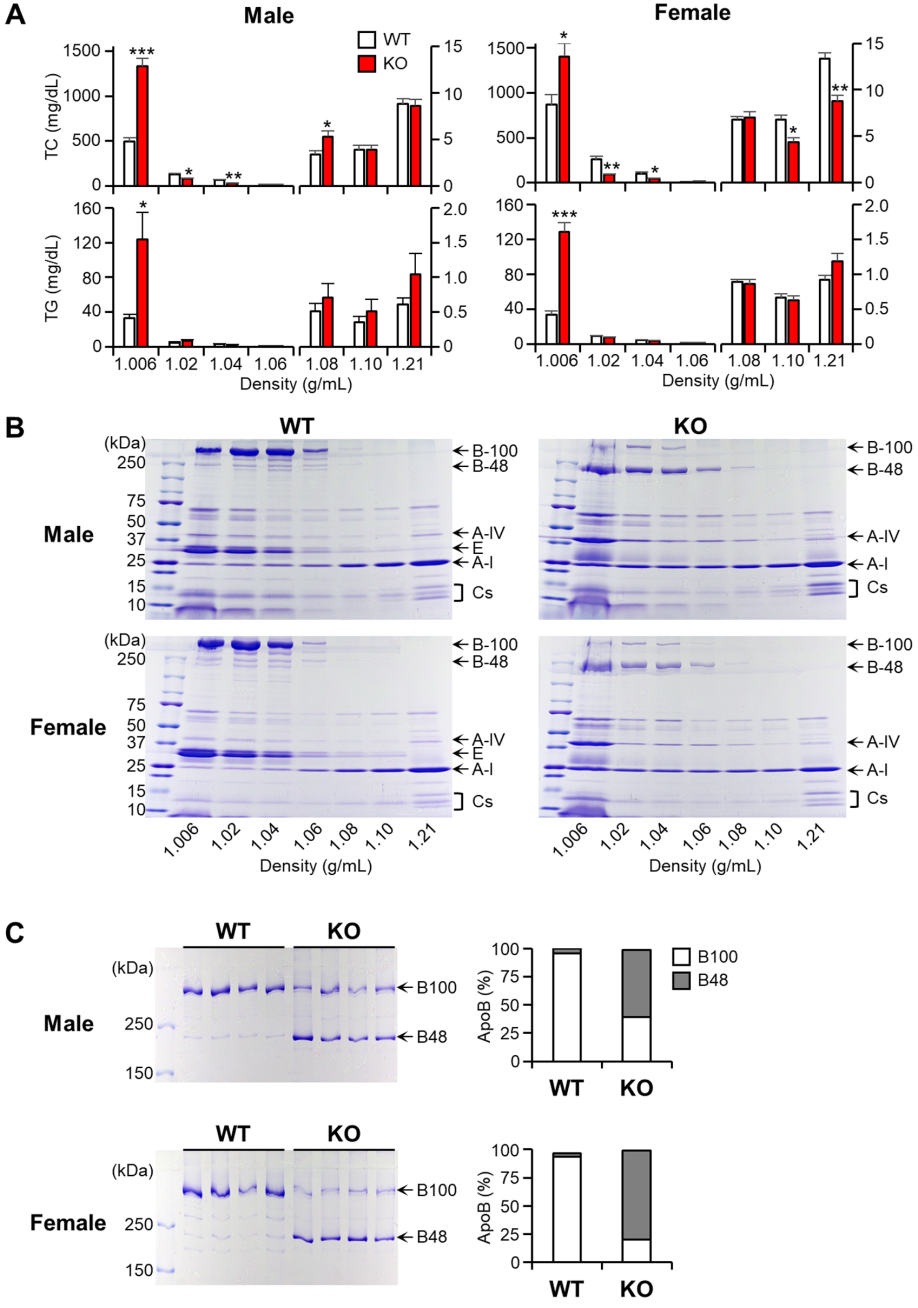
Analysis of plasma lipoproteins and apolipoproteins. Rabbits were subjected to a cholesterol-rich diet, and plasma lipoproteins were isolated into seven density fractions using ultracentrifugation. (**A**) The TC and TG contents were determined for each fraction. The data are presented as mean ± SEM, with a sample size of 4 per group. Statistical significance was indicated as follows: *p < 0.05, **p < 0.01, ***p < 0.001, when compared to the WT group. (**B**) The apolipoproteins were fractionated using 4–20% SDS-PAGE and subsequently stained with CBB. Representative gel images were provided, with each apolipoprotein indicated based on its protein size. (**C**) The fractions with density <1.006 g/ml were subjected to further analysis using 4% SDS-PAGE, and the ratio of apoB100 to B48 was calculated using an image analysis system. The data are presented as mean ± SEM, with a sample size of 4 per group.

Plasma lipoproteins were subjected to ultracentrifugation to separate them into seven density fractions, and the lipid contents of each fraction were measured and quantified (Figure 2A). In males, TC contents in *d* < 1.006 g/ml fractions (β-VLDLs) of apoE KO rabbits were significantly higher (2.7-fold↑ over WT), while IDL (37%↓) and large LDL (53%↓) were lower than those of WT rabbits. TG contents of KO rabbits were also significantly higher (3.7-fold↑ over WT) in β-VLDLs. As described above, female KO and WT rabbits had a similar levels of plasma TC (Figure 1A) but their distribution within lipoprotein fractions of KO rabbits were apparently different from WT rabbits: higher in β-VLDLs (1.6-fold↑ over WT) but lower in IDL (65%↓), large LDL (50%↓) and HDL (36%↓ in *d* = 1.10 and 34%↓ in *d* = 1.21 fraction). Similar to male rabbits, TG contents in β-VLDLs were significantly higher in female KO rabbits (3.8-fold↑ over WT).

To investigate the apolipoprotein components in each fraction, these proteins were fractionated by 4%–20% gradient SDS-PAGE and stained with CBB staining (Figure 2B). Notable differences observed on the SDS-PAGE gel were that in apoE KO rabbits, all apoB-containing particles (with densities <1.006, 1.02, 1.04, and 1.06 g/ml) were predominantly composed of apoB48, contrasting with the prevalence of apoB100 in WT rabbits (Figure 2B). This result was further strengthened by analyzing β-VLDLs using 4% SDS-PAGE followed by quantitating the ratio of apoB48/apoB100 in β-VLDLs using densitometry (Figure 2C). In apoE KO rabbits, apoB48 accounted for 59% in male and 79% in female of total apoB (apoB48 + apoB100), whereas in WT rabbits, apoB48 accounted for less than 5% in both males and in females. In addition, apoB protein levels of apoE KO rabbits were 1.2-fold higher (*p* < 0.05) in female and 1.3-fold higher (N.S.) in male than those of WT rabbits (Supplementary Figure S2).

### Electron microscopic analysis of apoB-containing lipoproteins

**Figure 3:**
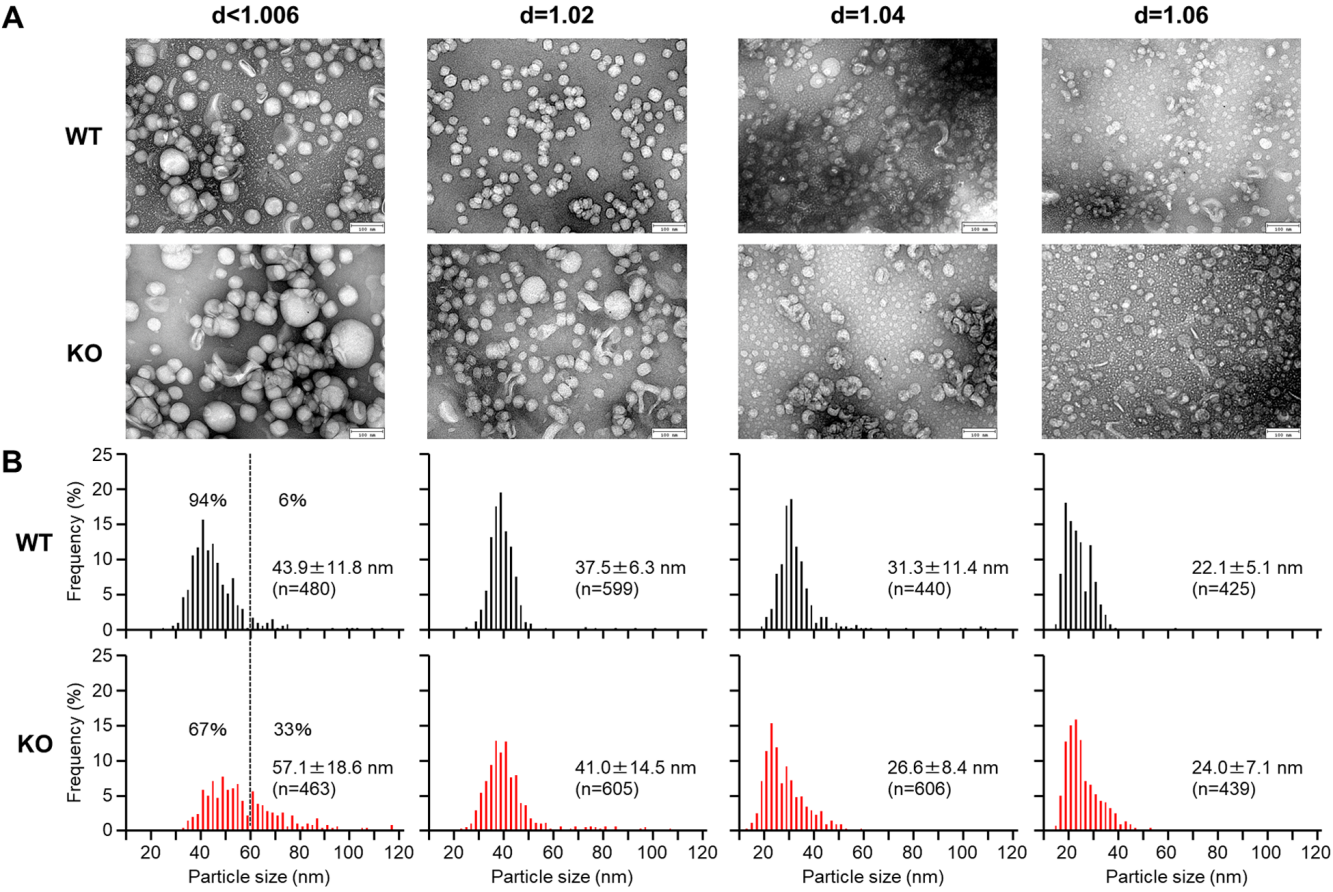
Electron microscopic analysis of apoB-containing lipoproteins. ApoB-containing lipoproteins (*d* < 1.006, *d* = 1.02, 1.04, and 1.06 g/ml) were isolated from male and female rabbits fed a cholesterol-rich diet. (**A**) The lipoproteins were stained with uranyl acetate and observation was made under a transmission electron microscope. (**B**) The diameter of the lipoprotein particles was measured. The data are presented as mean ± standard deviation (SD).

For evaluation of apoB-containing lipoprotein shapes and size, we examined these lipoproteins using transmission electron microscopy (Figure 3A). Apparently, the β-VLDLs (with density <1.006 g/ml) in apoE KO rabbits exhibited a higher abundance of large-sized particles with a diameter greater than 60 nm. These particles are likely to be CMs and chyloremnants of intestinal origin (as described above). These large-sized particles accounted for 33% of the total particles in apoE KO whereas only 6% in WT rabbits (Figure 3B). The mean diameter of *d* < 1.006 and *d* = 1.02 g/ml fractions was larger in apoE KO rabbits than those WT rabbits due to the increase of large-sized particles. The particle sizes of *d* = 1.04 and *d* = 1.06 g/ml fractions are similar in both apoE KO and WT rabbits (Figure 3B).

### ApoE KO rabbits had more atherosclerosis

**Figure 4:**
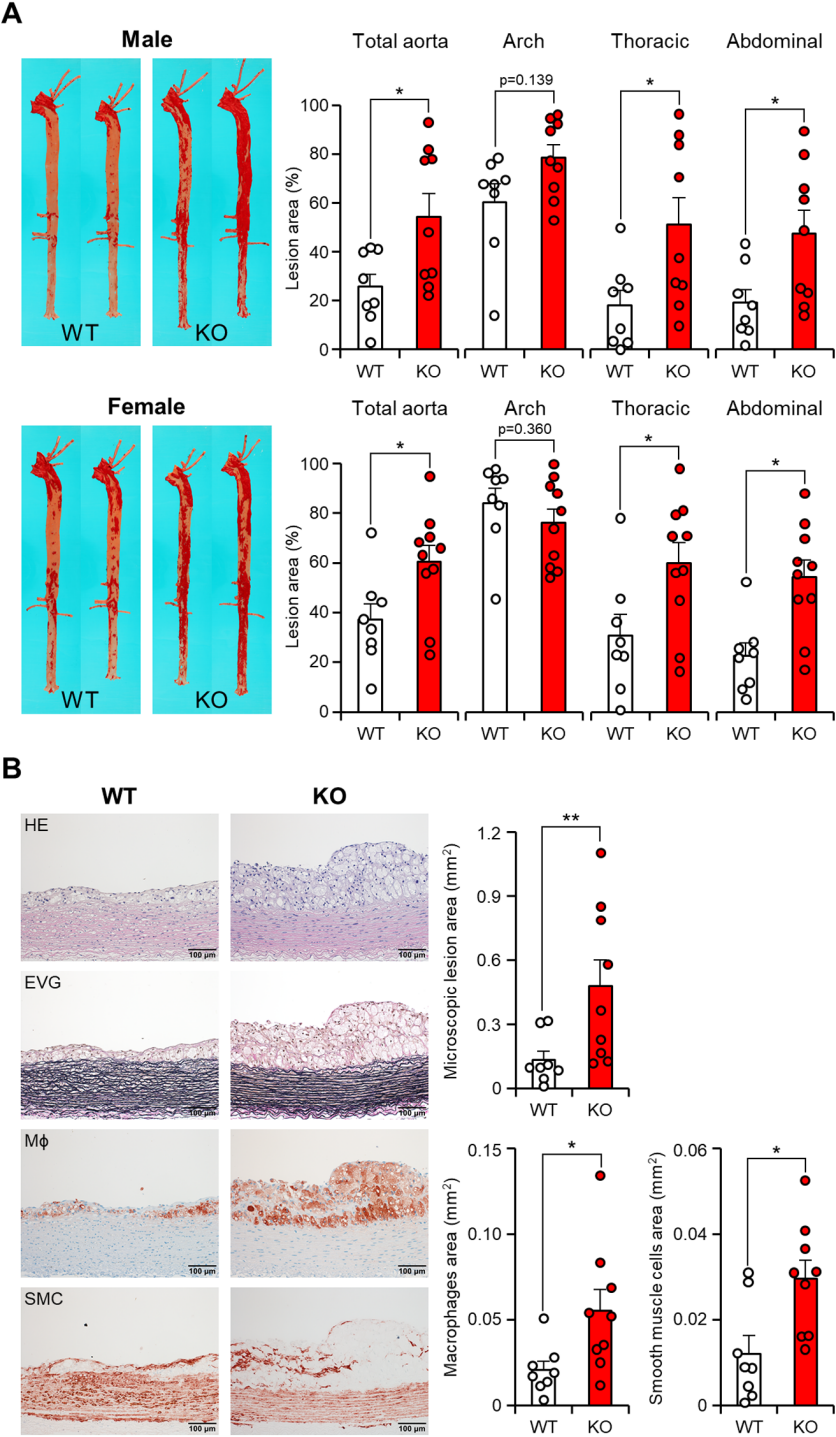
ApoE KO rabbits showed more atherosclerotic lesions. (**A**) Representative images of aortic trees stained with Sudan IV staining are shown on the left. The quantification of the Sudanophilic lesion area in each aortic segment was performed using an image analysis system and expressed as a percentage relative to the whole area. The data are presented as mean ± SEM, with a sample size of 8–10 per group. Statistical significance was indicated as *p < 0.05, compared to the WT group. Each dot represents an individual animal. (**B**) Microscopic analysis of the abdominal aortic lesions was conducted. Serial paraffin sections of the abdominal aorta were stained with hematoxylin and eosin (HE) and Elastica van Gieson (EVG), or immunohistochemically stained with monoclonal antibodies against macrophages (Mϕ) or α-smooth muscle actin for smooth muscle cells (SMC). Representative micrographs of each group are presented in the left panels. The microscopic lesion area and the area of immunohistochemically positive staining were quantified on the right panels. The data are presented as mean ± SEM, with a sample size of 8–9 per group. Statistical significance was indicated as *p < 0.05 and **p < 0.01, compared to the WT group.

In apoE KO rabbits, the *en face* lesion area of the entire aorta showed a significant increase (Figure 4A). Specifically, male apoE KO rabbits showed a 2.1-fold increase, while female apoE KO rabbits exhibited a 1.6-fold increase compared to the WT groups. Notably, this increase occurred despite the similar plasma TC levels observed in female rabbits compared to the WT rabbits. Comparison of various segments of the aorta showed that the enlargement of lesion size in apoE KO was predominantly driven by a significant increase in lesions within the thoracic aorta (2.8-fold increase in males and 1.9-fold increase in females) and the abdominal aorta (2.5-fold increase in males and 2.4-fold increase in females) when compared to their respective counterparts. However, the gross lesion areas in the aortic arch of apoE KO rabbits exhibited only a slight increase (1.3-fold increase in males and 0.9-fold increase in females) but not statistically significant (Figure 4A). Upon microscopic examination of the abdominal aortas, it was observed that both WT and apoE KO rabbits displayed atherosclerotic lesions characterized by the presence of varying quantities of macrophages and foam cells derived from macrophages, as well as smooth muscle cells, all intermixed with the extracellular matrix (Figure 4B, left). Quantification of microscopic lesions using image analysis system showed that apoE KO rabbits had greater intimal lesions: 3.6-fold more than WT rabbits. Immunohistochemical examinations showed that increased intimal lesions were due to 2.7-fold increase of intimal macrophages and 2.5-fold increase of smooth muscle cells (Figure 4B).

**Figure 5:**
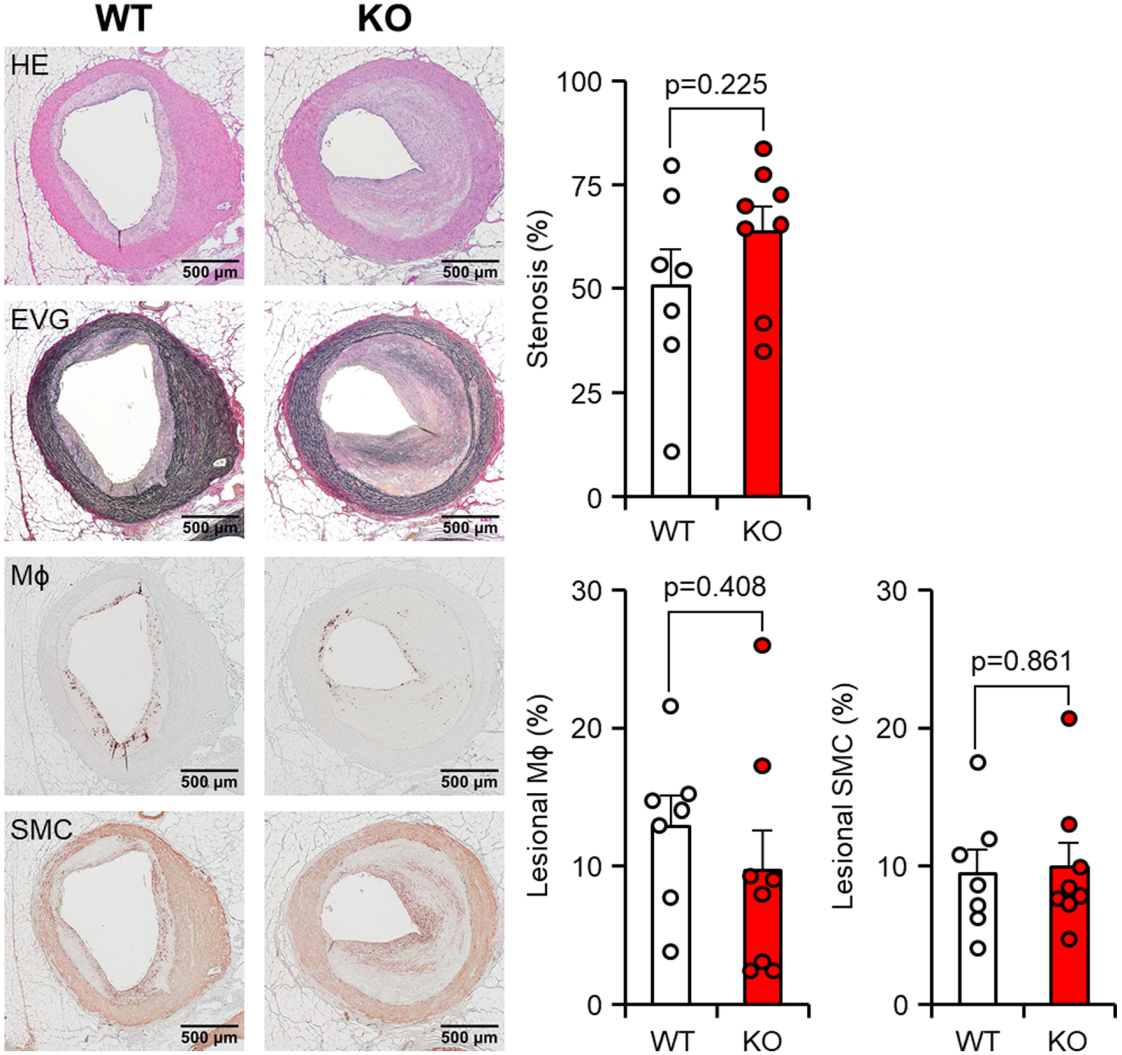
Microscopic analysis of the coronary atherosclerosis. Paraffin sections of the heart were then stained with hematoxylin and eosin (HE), Elastica van Gieson (EVG), or immunohistochemically stained using monoclonal antibodies against macrophages (Mϕ) or α-smooth muscle actin for smooth muscle cells (SMC). Representative micrographs of each group are displayed in the left panels. The degree of stenosis, lesional macrophages (Mϕ), and smooth muscle cells (SMC) were measured. Stenosis was calculated as a % relative to the lumen area of the coronary artery, which was defined by the internal elastic lamina. The areas occupied by Mϕ and SMC were expressed as a percentage relative to the intimal lesion area. The data are presented as mean ± SEM, with a sample size of 7–8 per group.

Given the fact that cholesterol-fed rabbits are known to develop coronary atherosclerosis, we were motivated to investigate the effect of apoE deficiency on the coronary atherosclerosis. In comparison to aortic lesions, coronary atherosclerotic lesions in both groups were found to have a more proportion of fibrotic tissues and a less abundance of cellular components (Figure 5, left). However, these lesions still resulted in varying degrees of luminal narrowing or stenosis. Quantification of the coronary stenosis rate showed that apoE KO rabbits exhibited mildly increased stenosis compared to WT rabbits with no significant difference (50.6 ± 8.7% in WT vs. 63.8 ± 6.0% in apoE KO, *p* = 0.225). There was no difference in the cellular components such as macrophages and smooth muscle cells in the coronary lesion (Figure 5, right).

**Figure 6:**
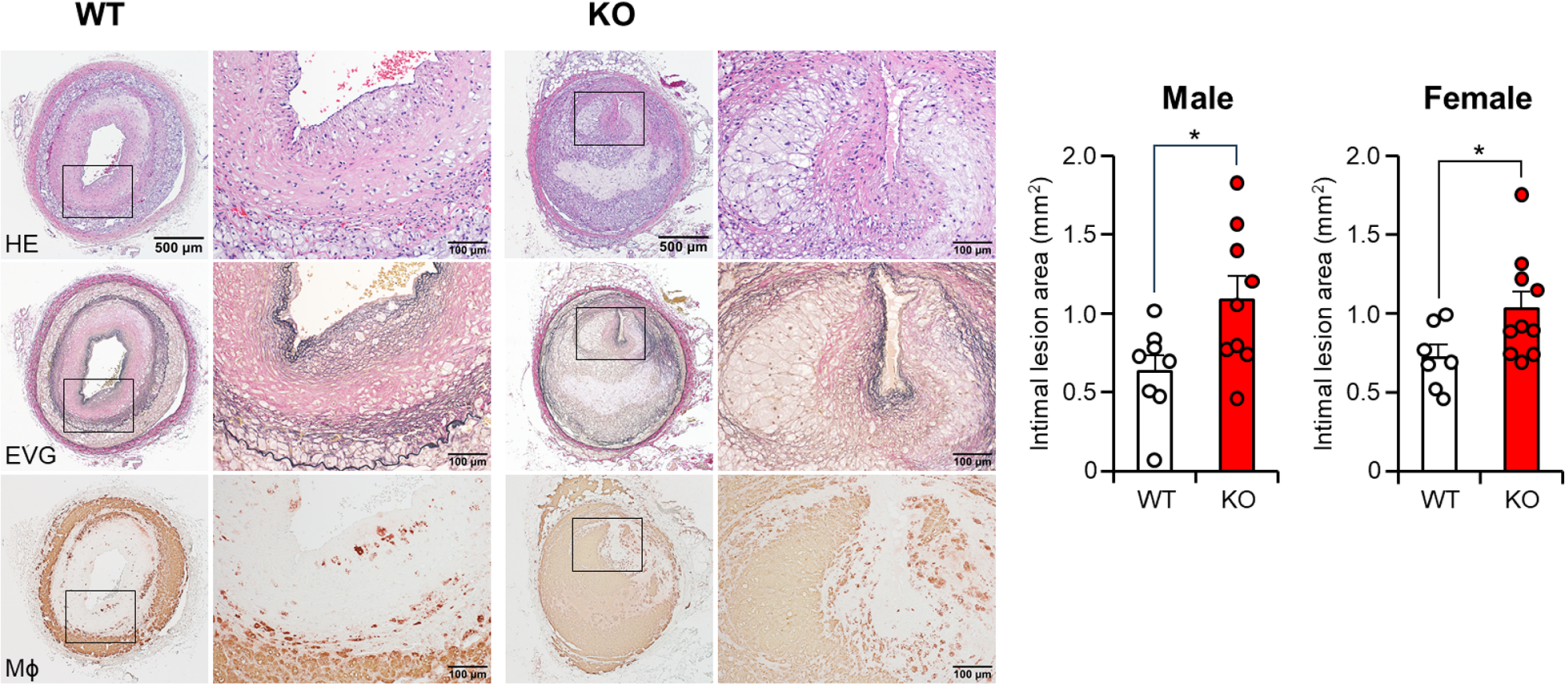
Femoral atherosclerosis induced by balloon injury. At the 5-week cholesterol-rich diet feeding, balloon endothelial denudation was induced in the right femoral artery. Rabbits were continuously subjected to a cholesterol-rich diet for a duration of 11 weeks. The femoral arteries with balloon-induced injury were embedded in paraffin. Subsequently, sections of the femoral arteries were stained with hematoxylin and eosin (HE), Elastica van Gieson (EVG), or monoclonal antibodies against macrophages (Mϕ). The intimal lesion area, as defined by EVG staining, was quantified and expressed in mm². The data are presented as mean ± SEM, with a sample size of 7–10 per group. Statistical significance was indicated as *p < 0.05 when compared to the WT group.

In addition to the spontaneous lesions induced by cholesterol diet feeding, we also examined whether apoE deficiency affects atherosclerosis induced by intimal injury. For this purpose, we generated balloon injury-induced atherosclerosis in the femoral arteries. The endothelial denudation of the femoral artery induced by balloon injury resulted in prominent neointimal thickening, which mainly consisted of smooth muscle cells, foam cells, and fibrotic tissue. The diameter of the balloon-injured femoral artery was observed to increase by 1.6-fold in both the WT and apoE KO groups when compared to the non-injured left femoral artery on the contralateral side (data not shown). However, apoE KO rabbits showed significantly greater intimal lesions than those of WT rabbits (*p* < 0.05). The atherosclerotic lesions of apoE KO rabbits were featured by more macrophages (Figure 6).

### ApoB48-rich remnant lipoproteins in apoE KO rabbits exhibited a higher susceptibility to copper-induced oxidation

**Figure 7:**
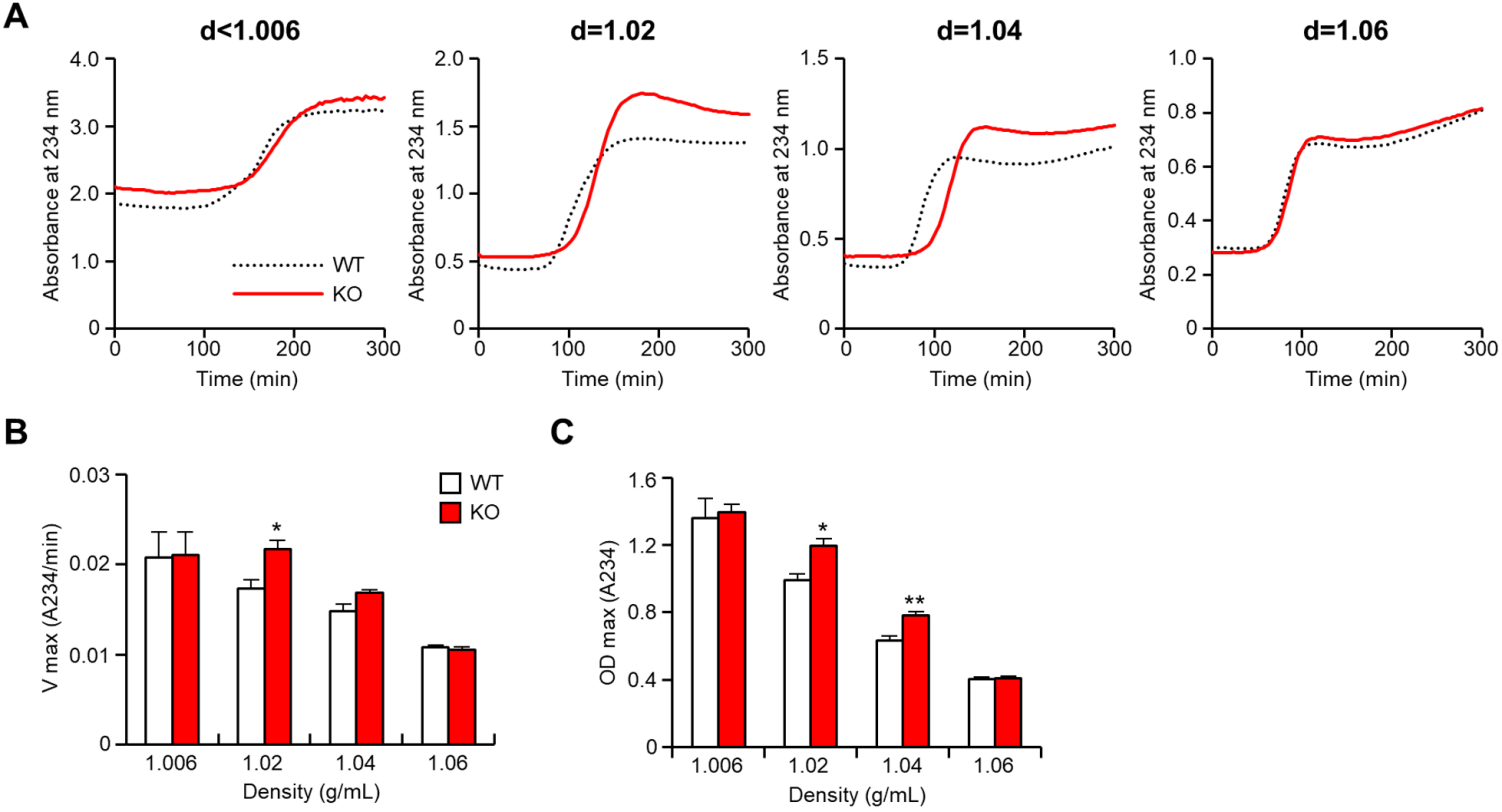
ApoB-containing lipoproteins from apoE knockout rabbits show enhanced oxidizability. ApoB-containing lipoproteins were isolated from rabbits fed a cholesterol-rich diet, and the oxidizability of these lipoproteins was assessed by monitoring lipid peroxidation through absorbance changes at 234 nm. (**A**) The representative dynamic alterations in absorbance were presented to illustrate the oxidation process. (**B**) The maximal oxidation speed (Vmax) of each apoB-containing lipoprotein was evaluated. (**C**) The maximal diene concentrations (OD max), indicative of lipid peroxidation, were displayed. The data are expressed as mean ± SEM, with a sample size of 4 per group. Statistical significance was indicated as *p < 0.05 and **p < 0.01, when compared to the WT group.

To evaluate the atherogenicity of remnant lipoproteins, we examined copper-induced oxidizability of the apoB-containing lipoproteins isolated from CRD-fed rabbits *in vitro* (Figure 7). The major atherogenic lipoproteins (*d* = 1.02 and 1.04 g/ml lipoproteins) of apoE KO rabbits exhibited apparently faster oxidation speed (Figure 7B) and conjugated diene production (Figure 7C) than those of WT rabbits, suggesting that these remnant lipoproteins were more susceptible to oxidization and presumably more atherogenic *in vivo*. The lag-time of the apoB-containing lipoproteins were not significantly difference in both groups (Supplementary Figure S3).

## Discussion

In this investigation, we aimed to assess the influence of apoE deficiency on hyperlipidemia and atherosclerosis by utilizing apoE KO rabbits as an experimental model. Rabbits are known to have lipid metabolism characteristics that are comparable to those observed in humans such as CETP and hepatic apoB editing (24). Under normal regular dietary conditions, apoE KO rabbits only exhibit mild elevation of plasma lipids, which is insufficient to induce atherosclerosis (25). Consequently, our study focused on examining their susceptibility to CRD-induced hyperlipidemia and subsequent atherosclerosis.

Male apoE KO rabbits exhibited more pronounced hypercholesterolemia and hypertriglyceridemia compared to their WT counterparts when fed a CRD. In contrast, female apoE KO rabbits showed a significant increase in plasma TG levels but had similar plasma TC levels as the WT control group, suggesting that males are more susceptible to diet-induce hyperlipidemia than females in the setting of apoE deficiency. The molecular mechanisms underlying the gender disparities in apoE functions or whether sex hormones play a role in mediating apoE functions remain unknown at present. However, similar observations have been reported in other genetically modified rabbit models (35, 36), suggesting that this phenomenon may have broader implications beyond apoE deficiency.

In spite of this, both male and female apoE KO rabbits showed marked increase of apoB48-rich remnant lipoproteins in plasma, which is in contrast to apoB100-rich remnant lipoproteins in WT rabbits fed a cholesterol diet. This finding indicates that apoE plays the major role in mediating the clearance of intestinally derived remnant lipoproteins. Unlike WT rodents (mice and rats), which are typically resistant to a cholesterol diet, WT rabbits are known to be sensitive to such a diet due to their lipoprotein metabolism features that resemble those of humans, as mentioned earlier. On cholesterol diet feeding, WT rabbits show elevation of plasma TC owing to the build-up of hepatically derived apoB-100 remnant lipoproteins whereas in the setting of apoE deficiency, hypercholesterolemia was further exacerbated owing to the increase of intestinally derived apoB-48 chyloremnants. Therefore, CRD-fed WT and apoE KO rabbits enabled us to compare the atherogenicity of apoB100-rich and apoB48-rich remnant particles *in vivo*.

ApoE KO rabbits exhibited a significant increase in aortic atherosclerosis when compared to WT controls. Histological and immunohistochemical examination showed that the enhanced lesions in apoE KO rabbits were primarily attributed to more infiltrating macrophages and proliferating smooth muscle cells. Furthermore, the enhancement of femoral atherosclerosis induced by balloon injury in apoE KO rabbits further indicates that regardless of the methods used, increased atherosclerosis was consistently observed in the context of apoE deficiency. In the current study, the susceptibility to atherosclerosis in apoE KO rabbits is highlighted in the aorta and femoral artery but only mildly increased in the coronary arteries. It is unclear why atherosclerotic burden differs from the distribution of arteries. It is known that the distribution of atherosclerotic lesions differs between familial hypercholesterolemia (FH) and type III hyperlipoproteinemia. Peripheral vascular disease of the lower extremities is almost as common as coronary artery disease in type III hyperlipoproteinemia. It is different from FH, in which there is less involvement of the lower extremities (10). In this study, we found that apoE KO rabbits, a model of type III hyperlipoproteinemia, showed more severe atherosclerosis in the aorta and femoral artery. Different types of hyperlipidemia may have different arterial preferences for atherosclerosis. Intracranial atherosclerosis of apoE KO rabbits was reported in previous studies (37, 38). Intracranial atherosclerosis was developed in apoE rabbits fed a CRD but WHHL rabbits, a model for human FH, exhibited more severe lesions than apoE KO rabbits.

The enhancement of atherosclerosis in apoE KO rabbits may involve several mechanisms. First, apoE deficiency leads to the impaired uptake of remnant lipoproteins in the liver, which results in the accumulation of these lipoproteins in the bloodstream and subsequently elevates plasma TC levels. Elevated plasma TC levels are a main atherogenic factor for the formation of atherosclerosis; therefore, this mechanism may be particularly relevant in male apoE KO rabbits, as they exhibit “higher” hypercholesterolemia compared to WT rabbits. However, this mechanism alone cannot fully explain the enhanced atherosclerosis observed in female apoE KO rabbits, as they exhibit similar TC levels to WT rabbits. Therefore, there may be additional factors or mechanisms at play that contribute to the enhanced lesion formation in female apoE KO rabbits.

The second possibility is that enhanced atherosclerosis was essentially caused by increased number of apoB48-rich remnant lipoproteins possibly independent on the plasma levels of TC because both male and female apoE KO rabbits possessed higher contents of apoB48. It was reported that apoE KO mice expressing only apoB48 developed more aortic atherosclerosis than apoE KO mice expressing only apoB100 (39). However, apoE KO mice expressing only apoB48 also had higher TC levels in plasma thus complicating the role of apoB48 in the development of atherosclerosis. Because female apoE KO rabbits had similar high hypercholesterolemia to WT rabbits, our results indicate that apoB48-rich remnant lipoproteins indeed are more atherogenic. ApoB48 is a protein that is primarily found in CMs and CM remnants, which are involved in the transport of dietary lipids from the intestines. Although it is generally considered that these particles are less atherogenic than apoB-100-containing particles because of their large size, our finding indicates that that these particles are indeed atherogenic.

To examine the hypothesis that apoB48-rich remnant lipoproteins are more atherogenic, we compared their oxidizability with those of apoB100-rich remnant lipoproteins *in vitro*. As shown in Figure 7, at the same amount of proteins, apoB48-rich remnant lipoproteins isolated from apoE KO rabbits exhibited a higher susceptibility to copper-induced oxidation than apoB100-rich remnant lipoproteins isolated from WT rabbits fed a cholesterol diet. The current *in vitro* oxidizability assay highlights the atherogenicity of *d* = 1.02 and 1.04 apoB-containing particles. However, the *d* < 1.006 particles did not show differences in oxidizability parameters between apoE KO and WT rabbits. The lack of difference may be related to lipid compositions and the amount of antioxidants for each lipoprotein particles. The lag-time, an antioxidant ability, of *d* < 1.006 fractions are higher than those of small apoB-containing particles (Supplementary Figure S3). Oxidative modification of lipoproteins is considered a critical event in atherogenesis (40, 41). Increased number of apoB48-rich remnant lipoproteins in plasma would certainly result in the enhancement of their deposition in the arterial wall and oxidization of these lipoproteins would subsequently lead to the monocyte adhesion, infiltration and foam cell formation (42, 43). It has been reported that poB48-rich remnant lipoproteins have a higher affinity for binding to receptors on arterial wall cells, such as macrophages and smooth muscle cells (44, 45). This increased uptake results in the accumulation of cholesterol and other lipids within these cells, forming foam cells, a hallmark of early atherosclerotic lesions (46). The build-up of apoB48-rich remnant lipoproteins in the intima triggers an inflammatory response as shown in our results that the lesions of atherosclerosis of apoE KO rabbits contain more macrophages. This response leads to the release of pro-inflammatory molecules further promoting the formation and progression of atherosclerotic plaques.

It is important to note that apoB48-rich remnant lipoproteins in apoE KO rabbits are not only enriched in cholesterol but also in triglycerides. Therefore, it is likely that the enhanced atherosclerosis observed in KO rabbits is partly attributed to elevated levels of TG. These lipid abnormalities, featured by increased levels of both TC and TG in the remnant lipoproteins, likely contribute to the development and progression of atherosclerosis in apoE KO rabbits. Many studies showed a causal role of triglyceride-rich remnant lipoproteins in the development of atherosclerotic cardiovascular disease (4, 47, 48). In addition, small-sized apoB48-containing particles were detected in postprandial plasma from hyperlipidemic subjects, and these particles were cleared more slowly and potentially increased coronary artery disease risk (49, 50).

In conclusion, our results strengthened the notion that apoB48-rich remnant lipoproteins are atherogenic. Therefore, reducing plasma CMs may become effective therapeutics for treating postprandial hyperlipidemia and cardiovascular disease. Understanding these processes not only helps shed light on the pathogenesis of atherosclerosis but also may offer potential targets for therapeutic interventions in the future.

## Data Availability

The raw data supporting the conclusions of this article will be made available by the authors, without undue reservation.
