# Supplementary material for: Enhanced atherosclerosis in apolipoprotein E knockout rabbits: role of apoB48-rich remnant lipoproteins
Source: Front Cardiovasc Med. 2024 Jul 17;11:1424064. doi: 10.3389/fcvm.2024.1424064 (PMC11288958; doi:10.3389/fcvm.2024.1424064)
Supplement: Supplementary file 1 [file Datasheet1.pdf]

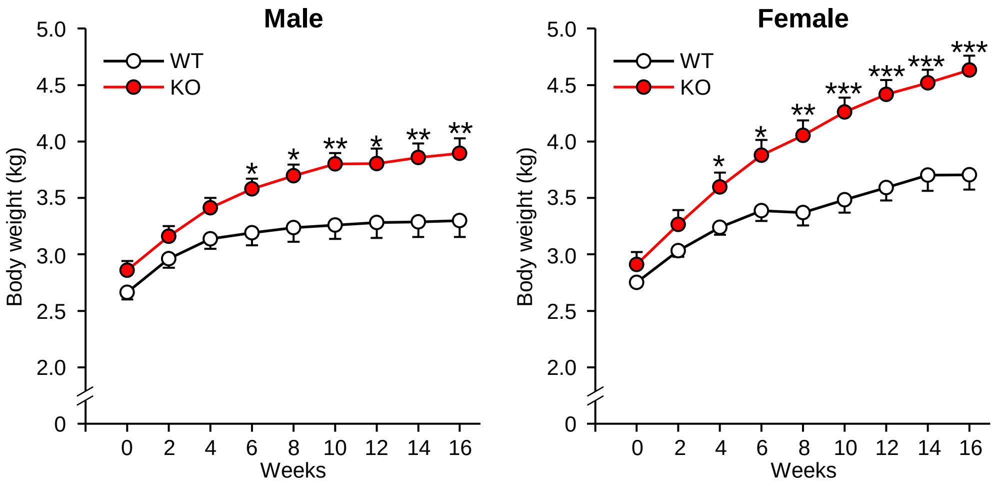

Supplementary Figure S1. The body weight changes of male (Left) and female (Right) rabbits. ApoE KO and WT rabbits were fed a cholesterol-rich diet for 16-week. The data are presented as mean ±SEM with a sample size of 8-10 per group. Statistical significance was denoted as follows: *p<0.05, **p<0.01, ***p<0.001, when compared to the WT group.

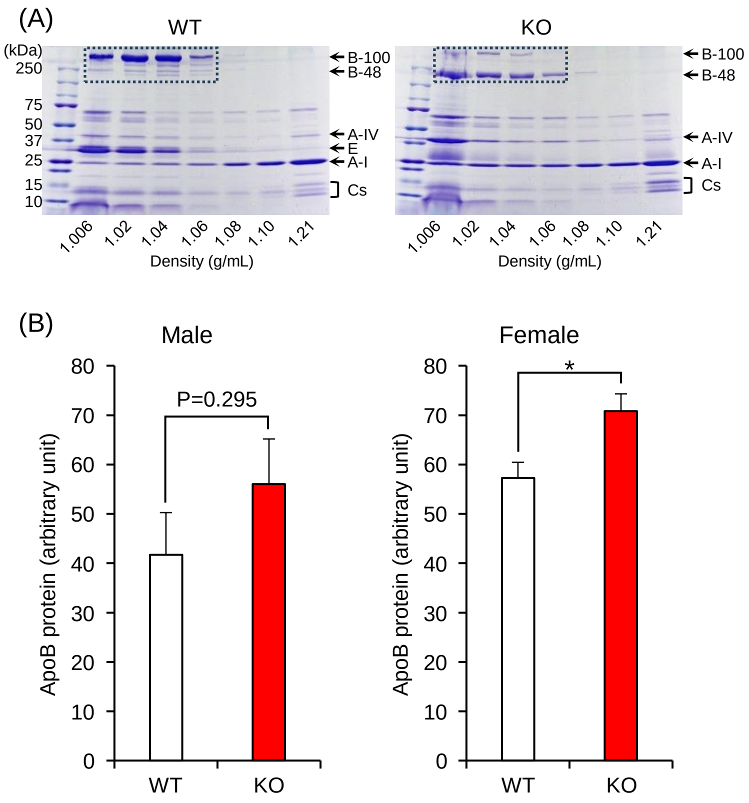

Supplementary Figure S2. Plasma apoB protein levels. (A) Method for densitometric quantification of apoB protein in d<1.006 –1.06 g/mL apoB-containing lipoprotein fractions. Using SDS-PAGE gel images with CBB-stained, the areas surrounded by the dashed line were quantified for apoB with NIH image software. ApoB relative values were obtained by normalizing CBB intensity with a standard protein. (B) ApoB protein levels of apoE KO and wild-type (WT) rabbits. The data are expressed as mean ±SEM, with a sample size of 4 per group. *p<0.05, when compared to the WT group.

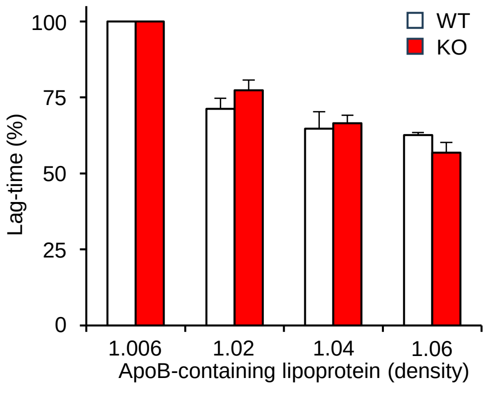

Supplementary Figure S3. Lag-time of apoB-containing lipoproteins of *in vitro* oxidizability assay. ApoB-containing lipoproteins were isolated from rabbits fed a cholesterol-rich diet, and the oxidizability of these lipoproteins was assessed by monitoring lipid peroxidation through absorbance changes at 234 nm. The data was shown as relative values with the lag-time of the d<1.006 fraction as 100%. The data are expressed as mean ±SEM, with a sample size of 4 per group.
